# Numerical cognition in speakers of an Amazonian language with exactly twenty number words

**DOI:** 10.1007/s00426-026-02300-x

**Published:** 2026-04-29

**Authors:** Vera da Silva Sinha, Wary Kamaiura Sabino, Silke M. Göbel, Asifa Majid

**Affiliations:** 1 Department of Experimental Psychology, University of Oxford, Oxford, UK; 2 Secretaria Estadual de Educação, Mato Grosso, Brazil; 3 Department of Psychology, University of York, York, UK

**Keywords:** Amazonian languages, Number words, Counting, Exact numerical cognition

## Abstract

Recent research has examined the relationship between number word vocabularies and performance on tasks requiring exact number representation and processing. Many studies in this area have focused on a small number of Amazonian languages, which are reported to have highly restricted number inventories, leaving other Amazonian systems comparatively under-documented. Here we investigate the Awetý number system and assess numerical performance of its speakers within the attested counting range for this language. Contrary to prior claims that Awetý counting is limited to five or ten terms, we show that Awetý has a shared conventional counting system consisting of twenty complex verbal expressions. Twelve Awetý speakers were tested using a battery of 13 tasks that assessed counting, verbal and non-verbal array matching, subtraction, and their Approximate Number System acuity. We compared Awetý speakers’ performance on non-verbal tasks to 12 speakers of Brazilian Portuguese. Awetý speakers’ performance was comparable to Brazilian Portuguese speakers across non-verbal number tasks. These findings show that Awetý speakers can reliably represent and operate on exact numbers up to 20, providing new empirical evidence from an under-described Amazonian language.

Humans and many other animals are widely considered to share the ability to perceive and discriminate small (3 or less) discrete quantities and larger approximate quantities (Dehaene, 1997; Feigenson et al., 2004; Schneider et al., 2022). Much recent debate about number cognition has focused on the role that language and symbolic notations plays in human species-specific number cognition, especially the representation and processing of exact large numbers (greater than 3 or 4) (Chrisomalis, 2021; Frank, 2024; Núñez, 2017). Cross-linguistic studies have often been used to explore the relationship between linguistic numeral systems and the representation and processing of exact numbers, including one-to-one matching and basic arithmetic operations.

Although restricted number systems are reported in many parts of the world (see, e.g., Calude, 2021), Amazonian languages have, in particular, received a lot of attention. It has been claimed that Amazonia can be recognised as a linguistic area based on features including: “only a small class of lexical numbers” (Dixon & Aikhenvald, 1999, p. 9), reflecting a widely held view that Amazonian languages typically exhibit numeral systems with less than five number words (e.g., Aikhenvald, 2015, 2022; Drude, 2009, reported in Chan, 2024; but see Silva Sinha et al., 2017). This has raised the question of whether non-linguistic numerical cognition is also restricted for speakers of these languages.

Much of the discussion of Amazonian number systems has centred on languages reported to have highly restricted numeral inventories, often framing the issue in terms of the absence versus presence of number words. At the same time, Amazonia is a region of exceptional linguistic diversity, and number systems vary considerably across languages (Dixon & Aikhenvald, 1999; Aikhenvald, 2015). Before broader theoretical claims about language and number cognition can be evaluated, there is a need for careful documentation of under-described systems and for examining how speakers perform on numerical tasks within their attested number lexicon range.

Initial evidence from the Pirahã people of Amazonian Brazil has been central to this debate. The Pirahã language has three quantifying words corresponding to ‘roughly one’, ‘roughly two’, and ‘many’ (e.g., Gordon, 2004; Everett, 2005; Frank et al., 2008). Gordon (2004) argued on the basis of non-linguistic matching tasks that Pirahã speakers did not represent exact quantities above three. Subsequent work challenged this interpretation suggesting that performance may depend on test structure and context (Frank et al., 2008; Everett & Madora, 2012). A parallel line of evidence comes from another Amazonian language that has contributed important evidence to this debate, Mundurukú, which also has been reported to have a small number word inventory (Pica et al., 2004). There are complex polymorphemic expressions for numbers up to 5 in the language, but speakers seem to use them approximately in naming. Although Mundurukú speakers performed above chance on non-verbal approximate number tasks and showed a typical ratio effect, their performance on tasks requiring exact number representation declined as set sizes increased. Taken together, these studies have been used to support the idea that the lexicalized number range may constrain exact number cognition, although there is continued debate around the exact roles of language, culture, and task demands (e.g., Casasanto, 2005; Gelman & Gallistel, 2004; Laurence & Margolis, 2007; Pica & Lecomte, 2008; Flaherty & Senghas, 2011; Spaepen et al., 2011; Cipora et al., 2023). The persistence of these debates points to the need for more fine-grained documentation of number systems and numerical performance.

A recent study of the Tsimane’ language of the Bolivian Amazon, which has a productive base-10 number system (e.g., ‘eleven’ is ‘ten-one’, ‘twelve’ is ‘ten-two’, etc.), sheds more light on the relationship between individual number inventory and exact numerical cognition (Pitt et al., 2022). Although the Tsimane’ number system is in principle capable of expressing quantities beyond 40, in practice not all speakers use or reliably count up to this range. Pitt et al. compared the performance of speakers with a personal inventory limited to around 20 number terms (“low-counters”) with that of speakers who can count up to 40 or beyond (“high-counters”). In simple matching tasks, both groups performed relatively well. However, as tasks increased in complexity, participants’ ability to make exact numerical matches was constrained by their highest verbal count. Low-counters, in particular, appeared to switch from exact to approximate strategies once quantities exceeded their individual highest verbal count range (Pitt et al., 2022).

Alternative perspectives have emerged in other cross-cultural contexts. For example, research by Butterworth et al., (2008) on Indigenous Australian children suggests that exact number understanding may develop even in contexts with limited formal mathematics instruction, emphasizing the role of domain-general cognitive mechanisms. Although these studies involve different populations and linguistic environments, they highlight ongoing debate concerning the relative contributions of language, education, and non-linguistic cognitive systems in numerical development.

Taken together, many studies suggest a relationship between an individual’s verbal number inventory and their performance on exact numerical tasks. There is, however, a need for further data from linguistic and cultural communities representative of a wider range of number systems. Accordingly, the current study provides new evidence from speakers of an under-described Amazonian language, Awetý, which we will show has exactly twenty number words. Specifically, we document the extent of the Awetý number system and assess speakers’ performance on verbal and non-verbal numerical tasks within this attested range (1–20).

Awetý (also known as Awetí, Awytyza, Enumaniá, Anumaniá, Auetö) is an indigenous community living in the centre of the Upper Xingu region of Mato Grosso state, Brazil. Awetý is a Tupian language whose closest linguistic relatives are those of the Tupi-Guarani family (Rodrigues & Cabral, 2012). Many Awetý individuals speak several languages, such as Kamaiurá and Aura, and the youngest increasingly speak Brazilian Portuguese. The population is about 365 people who live in five separate villages: Aldeia Awetý, Aldeia São Jorge, Aldeia Saidão/Fumaça, Aldeia Mirassol and Nyarazaul. Like other indigenous communities in Brazil, the Awetý have to fight for the preservation of their language and culture. The Awetý community today is the result of a fusion of two groups: the *Enumaniá* (also known as *Enumaniah*,* Anumaniá*) and the “true Awetý” (Sabino, 2016, pp. 18–29).

The economy is based upon small-scale subsistence agriculture, fishing, and gathering fruits. In addition to planting manioc and corn, Awetý also grow *papaia*, *urucu* (Achiote plant that produces annatto, a natural orange-red condiment), sweet potatoes, cotton (to make hammocks), and squash. The Awetý live in extended families in large communal houses distributed around an open space. Traditionally, men are responsible for hunting, fishing, and preparing the soil to plant crops, and also for producing ritual artefacts and hunting weapons, such as bows and arrows, and spears to fish. Women are responsible for harvesting crops, preparing and processing food, and collecting wood for fire. They also produce handcrafted products such as necklaces, bracelets, belts, and hammocks. Women also know how to process the *jenipapo*, a wild fruit that produces ink for body painting and decorating objects, especially pots (Silva Sinha, 2019). There is limited engagement with the national cash economy (e.g., some people selling handcrafts) but within the community, people mostly work with a bartering and exchange system.

In Awetý culture, time is based on events, meaning that time intervals are indexicalized by environmental happenings, the movements of the sun, the moon and the stars, and the regularities of social life and habitus (Silva Sinha, 2019, 2022). Temporal concepts are not metric (based on an ordinal enumeration of clock or calendar time intervals), not cyclical, and not represented in terms of a timeline. There are no words for weeks, months and years. The closest concept to year is referred to by the word *Kwarype*, meaning dry season, derived from the root meaning *Kwar* ‘sun’. People tend to translate it as ‘year’, comprised of two seasons, dry and rainy. The sun is central to the conceptualization of time intervals in both day and dry season, while water and rain are used for the rainy season intervals. Similarly, the moon and the absence of sunlight are used to name night intervals. There is no evidence of verbal counting or quantification of time intervals by numerals, although the use of a knotted string to estimate the duration of a planned fishing trip has been documented (Silva Sinha, 2019).

The only previously published description of the Awetý number system (Drude, 2009, reported in Chan, 2024) claims that it is limited to five or ten terms, with expressions for numbers from 1 to 10, but without consistency or stability for numbers above five. Specifically, it has been claimed that: “Aweti people can count up to ten, but the numbers from 6 to 10, and even 5, seem not to be much lexicalized, and are not often used anyway” (Drude, 2009, reported in Chan, 2024). The documentation of the above description is, however, sparse. In contrast, previous research by our team, based on linguistic and cultural fieldwork including native speaker knowledge, indicated that the Awetý number system and counting practices extend to at least twenty. This discrepancy was one motivation for the present study, which systematically investigated the extent of the Awetý number lexicon, the consistency of its usage and understanding by native Awetý speakers, and native Awetý speakers’ performance on linguistic and non-linguistic tasks designed to investigate their approximate and exact number cognition.

Our study therefore provides an empirical test of prior claims that Awetý counting is limited to five or ten terms, and investigates Awetý speakers’ performance on linguistic (in Awetý) and non-linguistic tasks within the exact number range 1–20. comparing their performance on the non-linguistic tasks with that of monolingual Brazilian Portuguese speakers. In addition, we assess approximate number cognition in both Awetý and Brazilian Portuguese speaking participants.

## Methods

### Participants

Twelve Awetý and twelve Brazilian Portuguese speakers participated in the experiment. The sample size was based on the availability of Awetý speakers willing to participate in the experiment; an equal number of Brazilian Portuguese speakers was sought. Awetý speakers (6 females, 6 males) were members of the Nyarazul Community (Xingu National Park, Mato Grosso State, Brazil). In Awetý society, age is not measured in years or months, so exact age is not known, but most participants were young adults (equal numbers of male and female) and two (one male, one female) were elderly (i.e., grandparents). As reported in the demographic questionnaire, none of the Awetý participants had received formal education outside their community. While some had attended local indigenous schools, their exposure to formal mathematics education was limited and not uniform across individuals. Educational provision within the community is locally organised and does not necessarily follow a standardised national curriculum, and mathematics instruction typically focuses on foundational arithmetic skills. All were bilingual or multilingual, as marriage between Awetý and other Indigenous and non-Indigenous individuals outside the Xingu area is common. Some participants also spoke other Indigenous languages from the Xingu region, including Kamaiurá, which has a lexicalized number system extending to 20 (Seki, 2000). Older people speak no or very little Brazilian Portuguese (although they speak other Indigenous languages); younger people speak a variety of Brazilian Portuguese (Portugues Indigena) that differs from standard Brazilian Portuguese in grammar and lexicon.

The 12 non-Indigenous Brazilian Portuguese speakers were all monolingual, age range = 20–78 years old (*M*_age_ 46 years, *SD* = 17.16 years), 5 females and 7 males. They were from different regions of Brazil (Rondônia, Acre and Mato Grosso). All had some level of formal education.

### Materials

Thirteen tasks were performed to assess numerical knowledge following a *Fieldwork manual.* Awetý and Brazilian Portuguese speakers were tested on their approximate number comparison abilities and six tests of number comprehension and production that only required non-verbal answers. One Brazilian Portuguese speaker did not participate in three tasks, i.e., approximate number comparison, delayed audio-visual matching (ascending and random condition), subtraction (three-alternative forced-choice 3AFC); another Brazilian Portuguese speaker did not participate in the subtraction (3AFC) task. In addition, only the Awetý speakers were tested on two additional tasks with non-verbal answers, and on four tasks that required them to respond with spoken number words. One Awetý speaker did not participate in five of these additional tasks.

## Approximate Number Comparison (ANS)

**Fig. 1:**
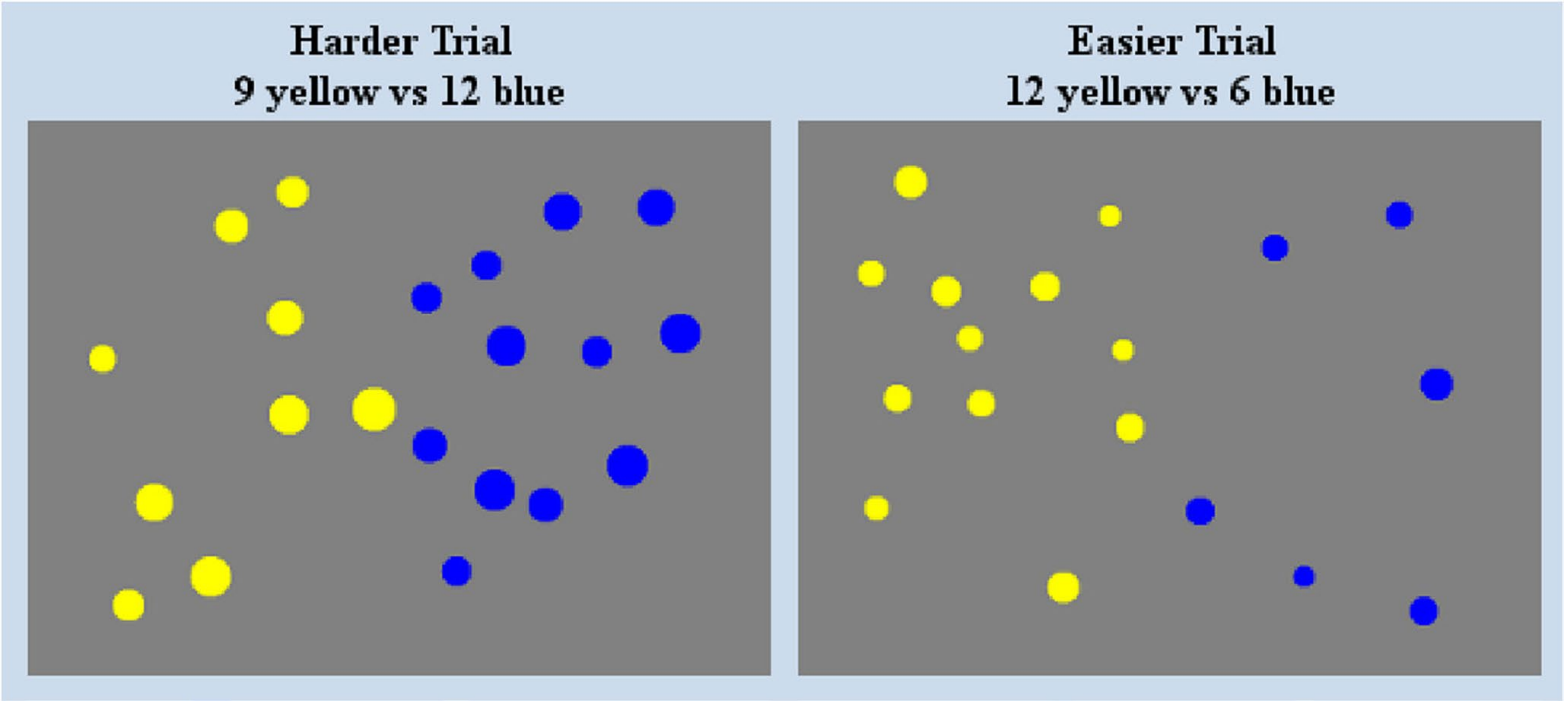
Examples of dot arrays used as stimuli in the approximate number comparison task, generated by the panamath software. These arrays are sample stimuli retrieved from the testing of a single participant

In order to assess participants’ ability to perform non-symbolic number comparison we used the Panamath task (Halberda et al., 2008). This computer-based task is assumed to measure the acuity of the Approximate Number System (ANS). Participants were presented with a set of blue and yellow dots in different quantities (range: 5 to 21 blue dots and 5 to 21 yellow dots, presentation time 600ms), that vary in ratio and total surface area, and participants had to judge on each trial whether there were more blue or yellow dots by pressing a button on a keyboard. Four different ratios (1.14, 1.24, 1.39, 2.39) were used. Trials varied in difficulty accordingly (see Fig. 1). Reaction times and accuracy of responses was recorded.

## Exact number comprehension, number production, and arithmetic with non-verbal responses

To examine exact numerical knowledge, participants engaged in a number of tasks that varied in their difficulty (following Frank et al., 2008; Flaherty & Senghas, 2011; Gordon, 2004); see Table 1 for an overview. We first describe materials and methods for tasks with non-verbal answers.

**Table 1:** Task overview

Tasks	Brief description	Groups tested
Approximate number processing		
PANAMATH Non-symbolic comparison (2AFC)	Computer-administered Panamath task (Halberda et al., 2008). Evaluates non-symbolic comparison ability by presenting different quantities of blue and yellow dots. Participants respond by 2AFC button press.	Awetý Brazilian Portuguese
Exact number understanding, number production and arithmetic		
Non-verbal responses		
Visual matching, linear array, ascending order	Wooden blocks presented in a line on the table, trials in ascending order from 1–20; participants have to reproduce the exact number using wooden blocks.	Awetý Brazilian Portuguese
Visual matching, linear array, random order	Wooden blocks presented in a line, trials in fixed random order as follows:14, 12, 10, 9, 11, 15, 6, 2, 5, 13, 1, 16, 7, 20, 3, 19, 8, 17, 4, 18; participants have to reproduce the exact number using wooden blocks.	Awetý Brazilian Portuguese
Visual matching, non-linear array, random order	Wooden blocks presented in a scattered array, trials in fixed random order as follows: 6, 10, 5, 12, 2, 17, 20, 18, 11, 1, 19, 4, 16, 9, 13, 15, 7, 3, 14, 8; participants have to reproduce the exact number using wooden blocks.	Awetý Brazilian Portuguese
Delayed audio-visual matching task, ascending order	Audio-recording of knocks of different numbers; participants have to reproduce the exact number using wooden blocks.	Awetý Brazilian Portuguese
Delayed audio-visual matching task, random order	Audio-recording of knocks of different numbers; trials in fixed random order as follows: 20, 10, 16, 9, 18, 3, 11, 5, 14, 19, 8, 2, 15, 1, 7, 12, 6, 13, 17, 4; participants have to reproduce the exact number using wooden blocks.	Awetý Brazilian Portuguese
Non-verbal subtraction (3AFC)	A set of A4 laminated cards featuring pictures of fruits in a basket. For a list of subtraction items and response options, see Table 4. Participants respond by pointing to one of three response options.	Awetý Brazilian Portuguese
How many (3AFC)	A set of A4 laminated cards featuring pictures of oranges, trials in fixed random order as follows: 18, 10, 15, 4, 2, 19, 6, 8, 13, 11, 14, 12, 17, 9, 1, 16, 20, 3, 7, 5; participants respond by pointing to one of three response options. For a list of response options, see Table 5.	Awetý
Give-N	The experimenter said a number between 1–20 in Awetý, trials in fixed random order as follows:18, 4, 8, 5, 13, 9, 15, 20, 7, 19, 1, 16, 6, 10, 17, 11, 3, 12, 2, 14; participants have to reproduce the exact number using wooden blocks.	Awetý
Verbal responses		
Sequential counting	Audio-recordings of differing numbers of knocks of different numbers, trials in fixed random order as follows:10, 18, 14, 8, 15, 6, 19, 9, 20, 16, 13, 4, 7, 12, 5, 17, 2, 3, 1, 11; participant responds verbally with a spoken number word.	Awetý
Subtraction	A set of laminated A4 cards displaying pictures of fish traps with fish swimming in and out; participant responds verbally with a spoken number word. For the list of subtraction items, see Table 4.	Awetý
How many	A set of A4 laminated cards featuring pictures of oranges. Trials in fixed random order as follows:10, 3, 14, 17, 11, 4, 16, 12, 1, 20, 13, 5, 2, 7, 18, 9, 15, 19, 8, 6; participant responds verbally with a spoken number word.	Awetý
Verbal counting	Participants were asked to count aloud from 1 up to the highest number they knew.	Awetý

### Visual matching

For three tasks, participants were presented with different numbers of wooden blocks and had to recreate the exact number of blocks. Participants were not required to produce or respond to a number word, instead they were asked to replicate the number of blocks shown in a sample array. Trials were time unrestricted, so participants were allowed to answer at their own pace, allowing them to employ exact counting strategies if they wished.

### Visual matching, linear array, ascending order

Participants sat at a table, facing the experimenter. The experimenter laid out a number of identical wooden blocks in a horizontal line on the table. After the blocks were laid out, they remained on the table as the participant was asked to match the quantity displayed. The instructions were: “Please, from your basket, take the wood blocks and lay them out in the matching quantity”. Participants completed 20 trials in ascending sequence of numbers from 1 to 20. Blocks were removed and reinstated across trials.

### Visual matching, linear array, random order

As above, but the presentation of wooden blocks in each trial was in a fixed random order (see Table 1 for more details).

### Visual matching, non-linear array, random order

As above, but now the stimuli were scattered in a cloud formation on the table instead of in a line. There was a fixed random order of presentation across trials.

### Delayed audio-visual matching

For another two tasks, participants heard a number of knocks and had to reproduce them with a visual display.

### Delayed audio-visual matching, ascending order

The experimenter played an audio-recording of a series of knocks, with trials presented in ascending order from 1 to 20. After the recording finished playing, the participant had to provide a wooden block for each knock. The instructions were: “Please, from your basket, take the woodblocks and lay them out on the table, matching the quantity of knocks that you just heard”.

### Delayed audio-visual matching, random order

As above, but the auditory stimuli were presented in a random order of knocks (see Table 1). After each trial, the experimenter removed all blocks before the next trial began, ensuring that no residual materials were visible between trials.

### Arithmetic: Subtraction task with verbal response (3AFC)

**Fig. 2:**
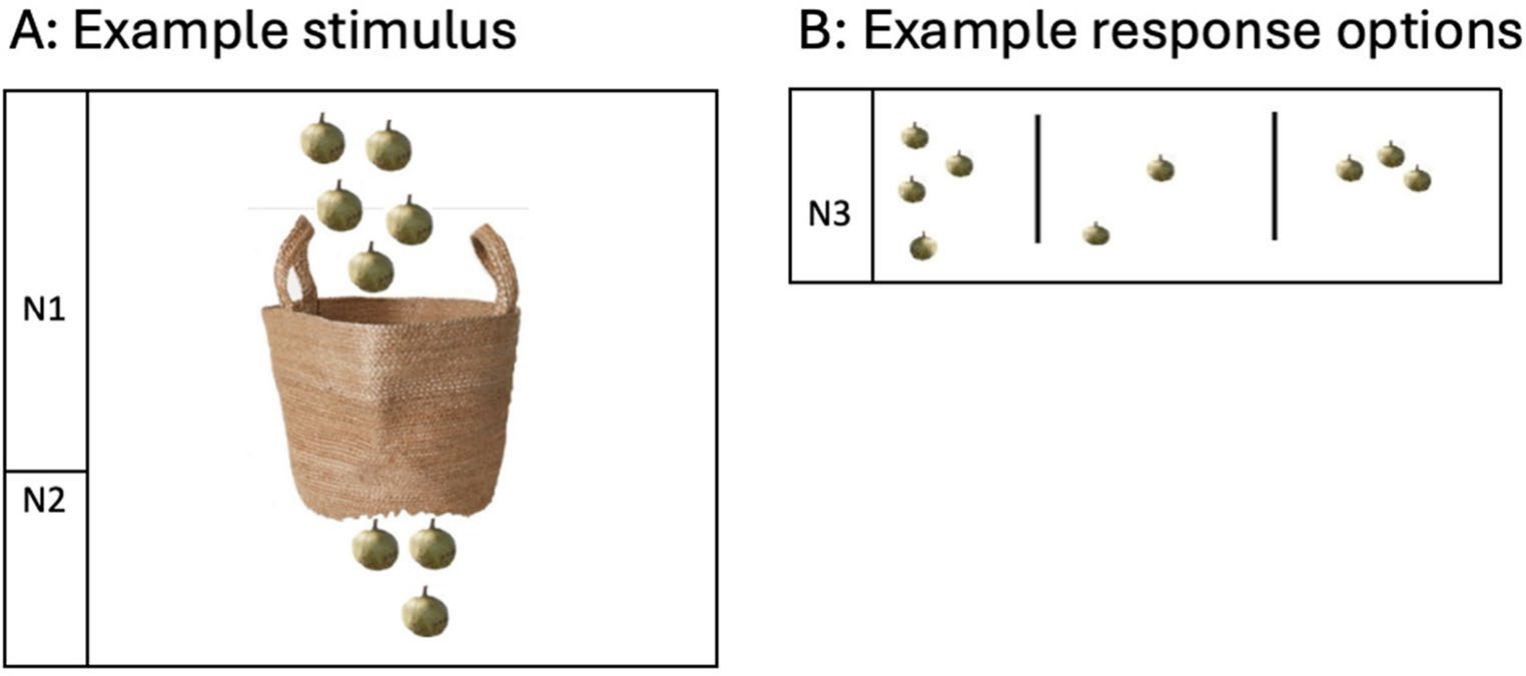
Panel **A **depicts an example stimulus for the non-verbal subtraction task with subtraction operands (N1, N2); Panel **B **depcits an example of response options (N3) for the non-verbal subtraction task

Participants were shown a picture of local fruit in a basket. They were told: “Here is a picture: I put N1 piqui in a basket, but N2 fell out. How many piqui are still left in the basket?” (see Fig. 2a). The number, N1, piqui fruit were pictured above the basket and N2 piqui below it. The participant had to select the correct answer from three randomly generated options displayed on the bottom of the same A4 laminated card by pointing to the correct array (3AFC; see Fig. 2b). For this task, operand quantities were selected from the range 1–20. Additionally, distractors were chosen to differ both from the correct answer and from each other, avoiding any unintended cues that could enable participants to solve the task without performing the subtraction (see Appendix Table 4 for a full list of items and distractors). The subtraction items were identical to those used in the Non-Verbal Subtraction (3AFC) task (see Table 4 for the full list of operands).

### How many, non-verbal response (3AFC)

**Fig. 3:**
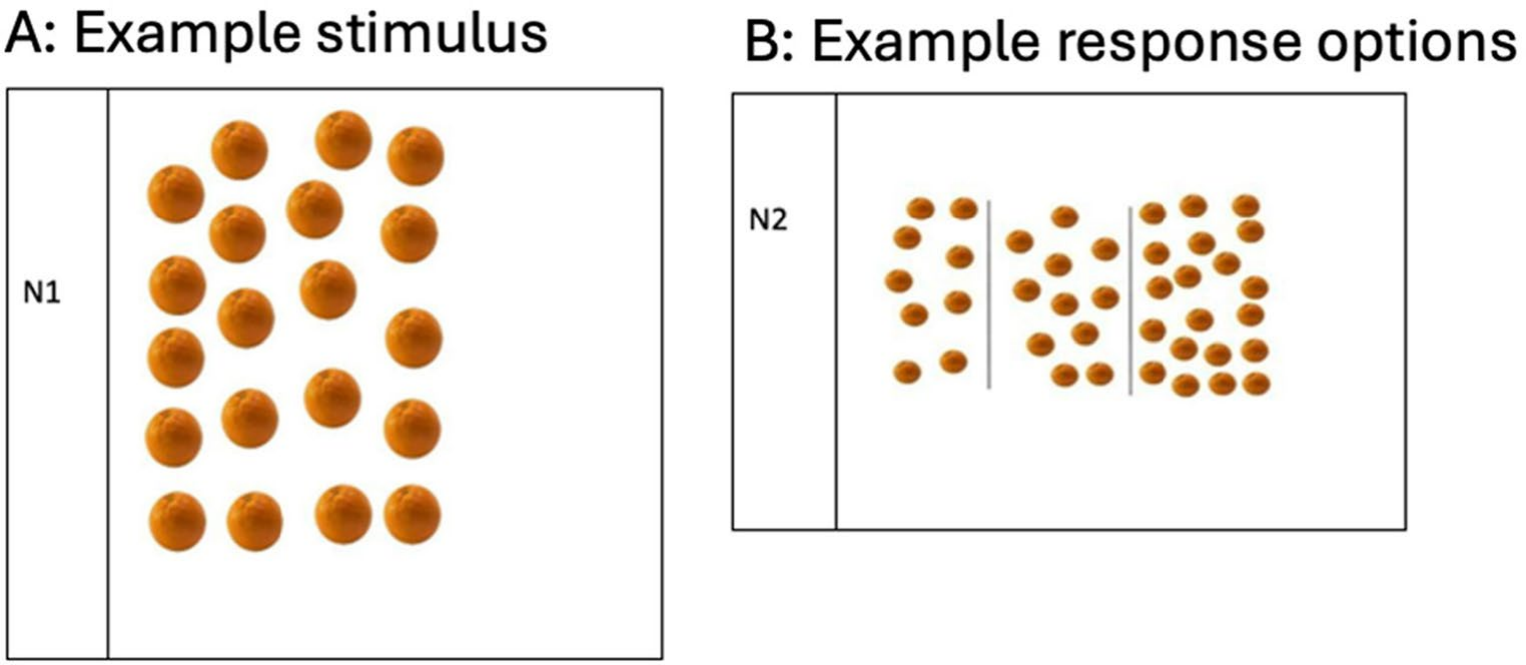
Panel **A**: gives an example target stimulus for How Many (non-verbal) (N1); Panel **B **gives an example of response options for How Many (non-verbal) (N2)

Participants saw a laminated picture card displaying *N* oranges (Fig. 3a) and were asked to indicate how many oranges they saw by pointing to one of three possible answers (Fig. 3b). Each trial was presented on a separate A4 laminated card displaying the target image (i.e., an array of oranges) at the top, and three numerical response options at the bottom. The target numerosity remained visible throughout the trial, and participants viewed all response options simultaneously. After the participant responded by pointing to one of the response options, the card was replaced with the next trial. The response options included distractors prepared in the same way as in the subtraction task (see Appendix Table 5 for full list of items and distractors).

### Give-N task

The experimenter said a number between 1 and 20 (random order) in Awetý, and participants were asked to give the exact number of wooden blocks corresponding to the stated quantity. This task tests participants’ understanding of the cardinal principle by assessing their ability to associate number words with specific set sizes. The exact instructions given were: “Please, from your basket, take the woodblocks and lay them out on the table, matching the quantity that you just heard”. This was a modified version of the Give-a-Number task (Wynn, 1990). The range 1–20 encompasses both previous published claims and field-based linguistic knowledge indicating that Awetý speakers use number expressions up to twenty.

## Exact number comprehension, number production, and arithmetic with verbal responses

To tap into verbal exact number knowledge in Awetý, various stimuli were presented only to Awetý participants, and verbal responses elicited in Awetý (following Marchand et al., 2022; Le Corre et al., 2006). Participants were required to produce the answer independently, encouraging a response generated in their native language without external cues.

### Sequential counting, random order

Participants heard an audio-recording presenting a randomly ordered series of sequences of knocks, and were asked to verbally state the exact quantity for each sequence in their native language. They were tested on all numbers from 1 to 20 to ensure a full assessment of their ability to accurately produce each number word within this range. Success on this task demonstrates the ability to produce the correct number word.

### Arithmetic: verbal subtraction

**Fig. 4:**
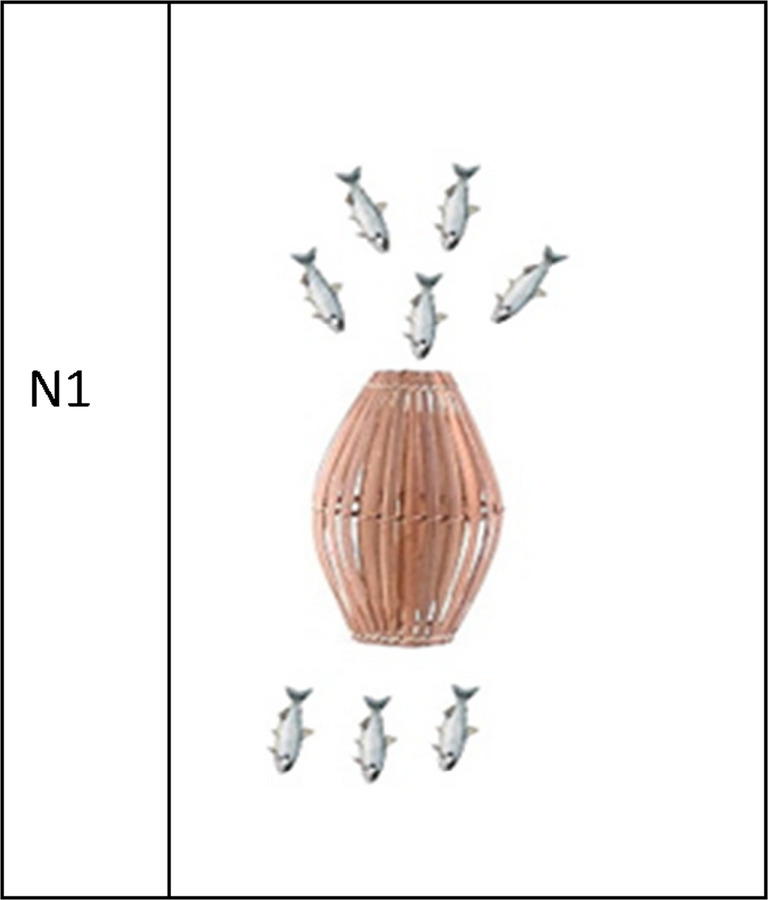
Example stimulus for the verbal subtraction task

Participants were shown a picture of a fish trap containing *N* fish (see Fig. 4), and were told: “Here is a picture of a fish trap. Some fish swim into the fish trap. And then some of them swim out again. How many are still in the fish trap?” Participants provided answers in their native language.

### How many, verbal response

Participants were presented with laminated picture cards of oranges (Fig. 3) and had to indicate how many oranges verbally in their native language, following these instructions: “Here is a picture of oranges. Can you please tell me how many oranges you see in this picture?”.

### Verbal counting

Awetý participants were asked to count from ‘one’ upward, as high as they could in their native language. Participants were allowed to use wood blocks (max *N* = 50), knots in a rope, or body parts to support their verbal counting, if they desired. This task focused solely on counting ability in Awetý to evaluate participants’ number knowledge within their native linguistic system.

## Procedure

All participants were tested individually with Awetý participants taking part in the study indoors in a communal house and Brazilian Portuguese participants in their home or garden. The first author conducted the study with the help of three native Awetý speakers, who were not participants in the study; Brazilian Portuguese data were collected by the first author alone. The three Awetý assistants supported the research process in designated roles to minimize potential influence on participants’ performance. One assistant operated the cameras to document each task accurately, another delivered all task instructions in Awetý, and the third assistant prepared materials for each task. The Awetý linguistic materials used in the tasks were developed and verified by the first and second authors. The second author is a native Awetý speaker, linguist and expert in Awetý language and culture. The first author personally supervised the tasks, ensuring consistency and accuracy in task administration. Prior to completing the experimental tasks, all participants completed a Demographic Questionnaire. For the Awetý participants, questions focused on whether participants had studied outside their communities, their experiences with mathematics education, and the languages they spoke. For Brazilian Portuguese speakers, the questionnaire included further questions about their education level and age. The study included both two-alternative forced-choice (2AFC) and three-alternative forced-choice (3AFC) tasks. Participants completed the numerical tasks described below (see Table 1).

## Results

Item level data for all participants and all tasks have been made available on OSF https://osf.io/hpvwq.

## Awetý number word knowledge

The study was designed in part to evaluate previous published claims (Drude, 2009, reported in Chan, 2024) suggesting that the Awetý number system is limited to five or ten terms, while also testing hypotheses informed by local linguistic and cultural expertise indicating that counting practices may extend to twenty. Accordingly, trials included both small (1–10) and larger (11–20) numerosities to assess the possible extent of participants’ number knowledge.- However, all Awetý participants demonstrated the ability to count up to 20 without hesitation. Thus, contrasts based on number size (small vs. large) became analytically irrelevant and are not reported. All Awetý speakers were asked whether they could count beyond twenty in Awetý, and all Awetý speakers said that this was not possible.

Awetý number words are morphologically complex. Pending a forthcoming detailed, usage-based linguistic treatment (Silva Sinha & Sabino Kamaiurá: Numeral construction and embodied quantification in the Awetý language, submitted), Table 2 provides an initial description of number expressions 1–20 based on current speaker-informed analysis.^1^

**Table 2:** Awetý number words with morpheme breakdown, English gloss, and free translation

#	Awetý number phrase	Morpheme-by-morpheme	English gloss by morpheme	Free translation
1	momozotsu	mo-mo-zo-tsu	hand-num.form-caus.com-similar	hand together
2	mokõj	mo-kõj	num.form-pair	pair (a pair or twins)
3	mojtaryka	mo-jtar-yka	num.form-rel1-without	a [pair] without a side
4	mokõj mokõj	mo-kõj mo-kõj	num.form-pair num.form-pair	pair pair
5	momozotsu kajpo pap	mo-mo-zo-tsu kaj-po pap	hand-num.form-caus.com-similar 3.incl hand comp	hand together is complete
6	momozotsu kajpo ytatap	mo-mo-zo-tsu kaj-po y-t-atap	hand-num.form-caus.com-similar 3.incl hand river-rel4-cross	(it) crossed, went over to the other side
7	mokõj kajpo ytatap	mo-kõj kaj-po y-t-atap	num.form-pair 3.incl hand river-rel4-cross	pair [fingers of] our hand crossed, went over to the other side
8	mojtaryka kajpo ytatap	mo-jta-yka kaj-po y-t-atap	num.form-rel2-without 3.incl hand river-rel4-cross	a [pair] without a side crossed, went over to the other side
9	mokõj mokõj kajpo ytatap	mo-kõj mo-kõj kajpo y-t-atap	num.form-pair num.form-pair 3.incl hand river-rel4-cross	pair pair crossed, went over to the other side
10	kajpo pap	kaj-po pap	3.incl hand comp	our hand[s] are complete
11	momozotsu kaipy ete oto	mo-mo-zo-tsu kai-py ete o-to	hand-num.form-caus.com-similar 3.incl-foot rel1 3-go	hand [toe of] our foot goes
12	mokõj kaipy ete oto	mo-kõj kai-py ete o-to	num.form-pair 3.incl-foot rel1 3-go	pair [toes of] our foot go
13	mojtaryka kaipy ete oto	mo-jta-yka kai-py ete o-to	num.form-rel1-without 3.incl-foot rel 3-go	pair one alone [toes of] our foot go
14	mokõj mokõj kaipy ete oto	mo-kõj mo-kõj kai-py ete o- to	num.form-pair num.form-pair 3.incl-foot rel1 3-go	pair pair of [toes of] our foot go
15	momozotsu kaipy opap	mo-mo-zo-tsu kai-py opap	hand-num.form-caus.com-similar 3.incl-foot comp	hand together foot completed
16	momozotsu kaipy weizo ytatap	mo-mo-zo-tsu kai-py wei-zo y-t-atap	hand-num.form-caus.com-similar 3.incl-foot 3-caus.com river-rel4-cross	hand together [toe of] our foot, crossed, went over to the other side
17	mokõj kaipy weizo ytatap	mo-kõj kai-py wei-zo y-t-atap	num.form-pair 3.incl-foot 3-caus.com river-rel4-cross	pair [toes of our] foot crossed, went over to the other side
18	mojtaryka kaipy weizo ytatap	mo-jta-yka kai-py wei-zo y-t-atap	num.form-rel2-without 3.incl-foot 3-caus.com river-rel4-cross	pair one alone [toes of] our foot crossed, went over to the other side
19	mokõj mokõj kaipy weizo ytatap	mo-kõj mo-kõj kai-py wei-zo y-t-atap	num.form-pair num.form-pair 3.incl-foot 3-caus.com river-rel4-cross	pair pair [toes of] our foot crossed, went over to the other side
20	kaipy opap	kai-py o-pap	3.incl-foot 3-comp	our feet are complete

The table and abbreviations follow Leipzig Glossing Rules, with minor adaptations for use with Awetý. Abbreviations: 1 (first person), 3 (third person), caus (causative), com (comitative), COMP (completive aspect), INCL (inclusive, first person plural including addressee), NUM.FORM (numeral formative), SIMIL (similative), WITHOUT (negated possession or absence). Relational Prefixes (REL1–REL4) mark the syntactic relationship between a noun and its determiner (based on Sabino, 2016:70–73): REL (in relation to) REL1 (determiner is contiguous with noun), REL2 (determiner is non-contiguous), REL3 (determiner is non-contiguous but coreferential with the subject), and REL4 (noun is generic and human)

### Approximate number processing

We analysed the accuracy and reaction times in the approximate numerical comparison task, using two separate groups (Awetý, Brazilian Portuguese) by ratio (1.4, 1.24, 1.39, 2.39) using ANOVA.

#### Accuracy

Both groups were relatively accurate (% correct), Awetý group *M*_Awetý_= 80.52, *SD*_Awetý_ = 7.20; Brazilian Portuguese group *M*_Brazilian Portuguese_ = 84.38, *SD*_Brazilian Portuguese_ = 6.56. There was no statistical difference between groups, *F*(1, 22) = 1.879, *p* = 0.184, *η*_*p*_^*2*^ = 0.079. However, accuracy was affected by differences in the ratio between the number of yellow and blue dots, *F*(3, 66) = 106.958, *p* < 0.001, *η*_*p*_^*2*^ = 0.829, with harder ratios leading to lower accuracy than easier ratios (ratio effect). There was no significant interaction between group and ratio effects, *F*(3, 66) = 1.484, *p* = 0.227, *η*_*p*_^*2*^ = 0.063.

**Fig. 5:**
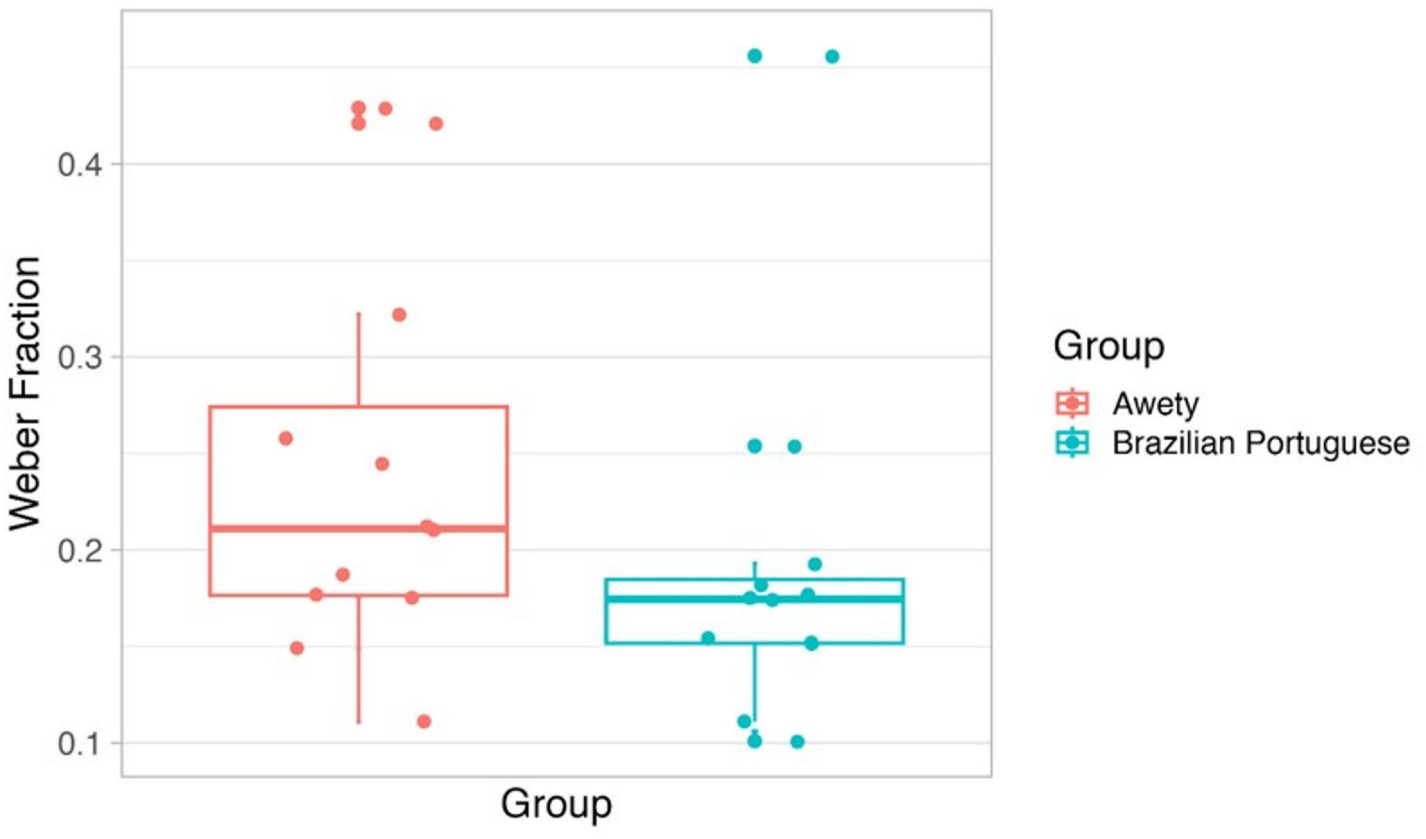
Box plot of the Weber Fraction for the approximate number processing task for both Awetý (red) and Brazilian Portuguese (blue) speakers; dots depict individual observations

The Weber fraction for the Awetý group ranged from 0.111 to 0.429 (*M*_Awetý_*=* 0.241, *SD*_Awetý_ = 0.101), and in the Brazilian Portuguese group, from 0.101 to 0.456 (*M*_Brazilian Portuguese_ = 0.190, *SD*_Brazilian Portuguese_ = 0.093) (see Fig. 5). Lower Weber fractions indicate a more precise, i.e. more accurate, performance. While the mean Weber fraction in the Brazilian Portuguese group was lower, this difference failed to reach significance, *t*(22) = 1.294, *p* = 0.209, Cohen’s *d* = 0.097. Large inter-individual differences in the Weber fraction can be observed in both groups.

#### Reaction time

The average reaction times (in ms) for Awetý speaking participants (*M*_Awetý_ = 1517, *SD*_Awetý_ = 585) were observed to be longer than those of Brazilian Portuguese speakers (*M*_Brazilian Portuguese_ = 1171, *SD*_Brazilian Portuguese_ = 281), although this disparity did not reach statistical significance, *F*(1, 22) = 3.412, *p* = 0.078, *ηp²* = 0.134. Overall, reaction time was significantly influenced by the ratio of yellow to blue dots, *F*(3, 66) = 9.165, *p* < 0.001, *ηp²* = 0.294. Furthermore, the interaction between groups and ratio was found to be statistically significant, *F*(3, 66) = 3.392, *p* = 0.023, *η*_*p*_^*2*^ = 0.134. Further examination of the effect of ratio on reaction times, conducted separately for each group using one-way repeated measures ANOVA, revealed that ratio significantly affected reaction times only for the Awetý speakers, *F*(3,33) = 9.551, *p* < 0.001, *ηp²* = 0.465. Conversely, there was no significant effect of ratio on reaction times in the Brazilian Portuguese speakers, *F*(3, 33) = 0.959, *p* = 0.423, *ηp²* = 0.080.

#### Summary

While response times were longer for the Awetý participants, there was no difference between the groups in their accuracy and their average Weber fractions. This suggests that Awetý participants have approximate number system cognition comparable to that of speakers of Brazilian Portuguese group.

### Exact number comprehension, number production, and arithmetic

Table 3 provides an overview of the results for all exact numerical tasks. Because the accuracy in nearly all tasks was unexpectedly high for the Awetý participants, we analyzed group differences using a non-parametric test for independent samples (Mann-Whitney) using JASP 0.18.1 (JASP Team, 2023). Visual inspection of the data suggests slightly greater variability for some mid-range numerosities (approximately 13–18). Given the small sample size and overall high accuracy, we refrain from drawing firm conclusions about this. Future research with larger samples would be needed to determine whether this pattern reflects systematic task-related demands or random variation. We also report the Bayes Factors for the independent samples Mann-Whitney U-Test (Goss-Sampson, 2020).

**Table 3:** Accuracy levels for exact task by language group

Task	AWETÝ				BRAZILIAN PORTUGUESE				Effect size			
	*N*	Mean (SD)	Min	Max	*N*	Mean (SD)	Min	Max	U^a^	*p* ^b^	rg^c^	BF_10_
***Non-verbal answers***												
Visual matching												
linear ascending	12	0.967 (0.049)	0.850	1.000	12	0.988 (0.031)	0.900	1.000	54.00	0.103	−0.250	0.917
linear random	12	0.963 (0.071)	0.750	1.000	12	0.983 (0.025)	0.950	1.000	64.00	0.305	−0.111	0.591
non-linear random	12	0.946 (0.040)	0.900	1.000	12	0.975 (0.040)	0.900	1.000	43.00	0.038	−0.403	1.531
Audio-visual matching												
ascending	12	0.917 (0.094)	0.750	1.000	11	0.968 (0.060)	0.850	1.000	43.00	0.063	−0.348	1.186
random	12	0.875 (0.106)	0.600	1.000	11	0.932 (0.119)	0.650	1.000	32.50	0.019	−0.508	2.244
Subtraction (3 AFC)	12	0.968 (0.082)	0.714	1.000	10	0.991 (0.020)	0.953	1.000	56.00	0.376	−0.067	0.566
How many (3 AFC)	12	0.971 (0.05)	0.850	1.000								
Give-N	11	0.973 (0.034)	0.900	1.000								
***Verbal answers***												
Sequential counting random	11	0.864 (0.148)	0.450	1.000								
Subtraction	11	0.922 (0.081)	0.714	1.000								
How many	11	0.950 (0.055)	0.850	1.000								
Verbal counting	11	1.000 (0)	1.000	1.000								

Notes:

**a** Mann Whitney.

**b** Bonferroni- corrected significance level is 0.007, independent sample, one-tailed,

**c** *rg* = glass rank biserial correlation coefficient

#### Non-verbal answers

**Fig. 6:**
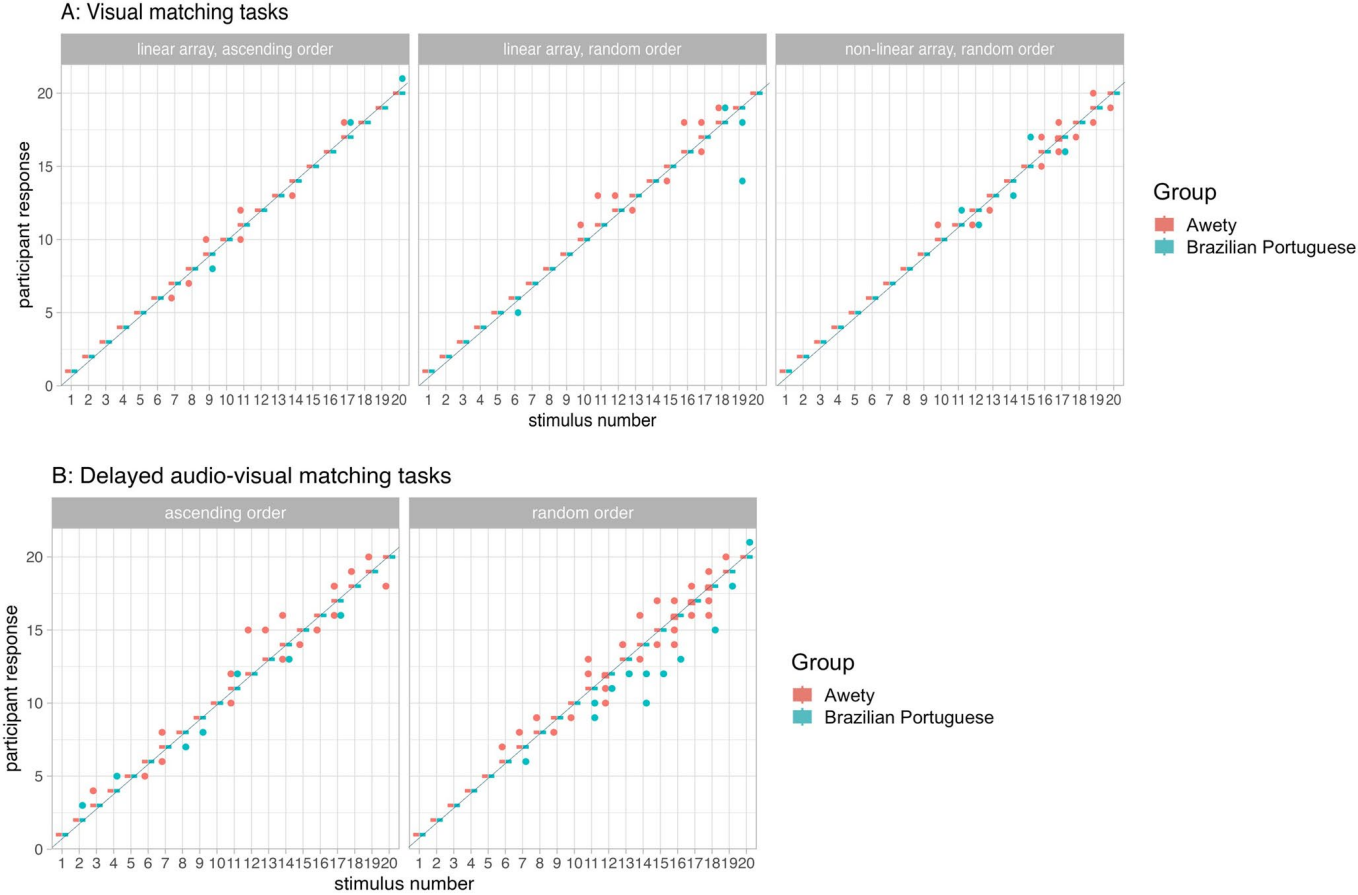
Box plots of stimulus number (x-axis) and participant response (y-axis) given by Awetý (red) and Brazilian Portuguese (blue) speakers, with individual responses (dots). Panel **A **depicts the visual matching tasks for linear array ascending order (left), linear array random order (middle), non-linear array random order (right). Panel **B **depicts the delayed audio-visual matching tasks for ascending order (left) and random order (right)

**Fig. 7:**
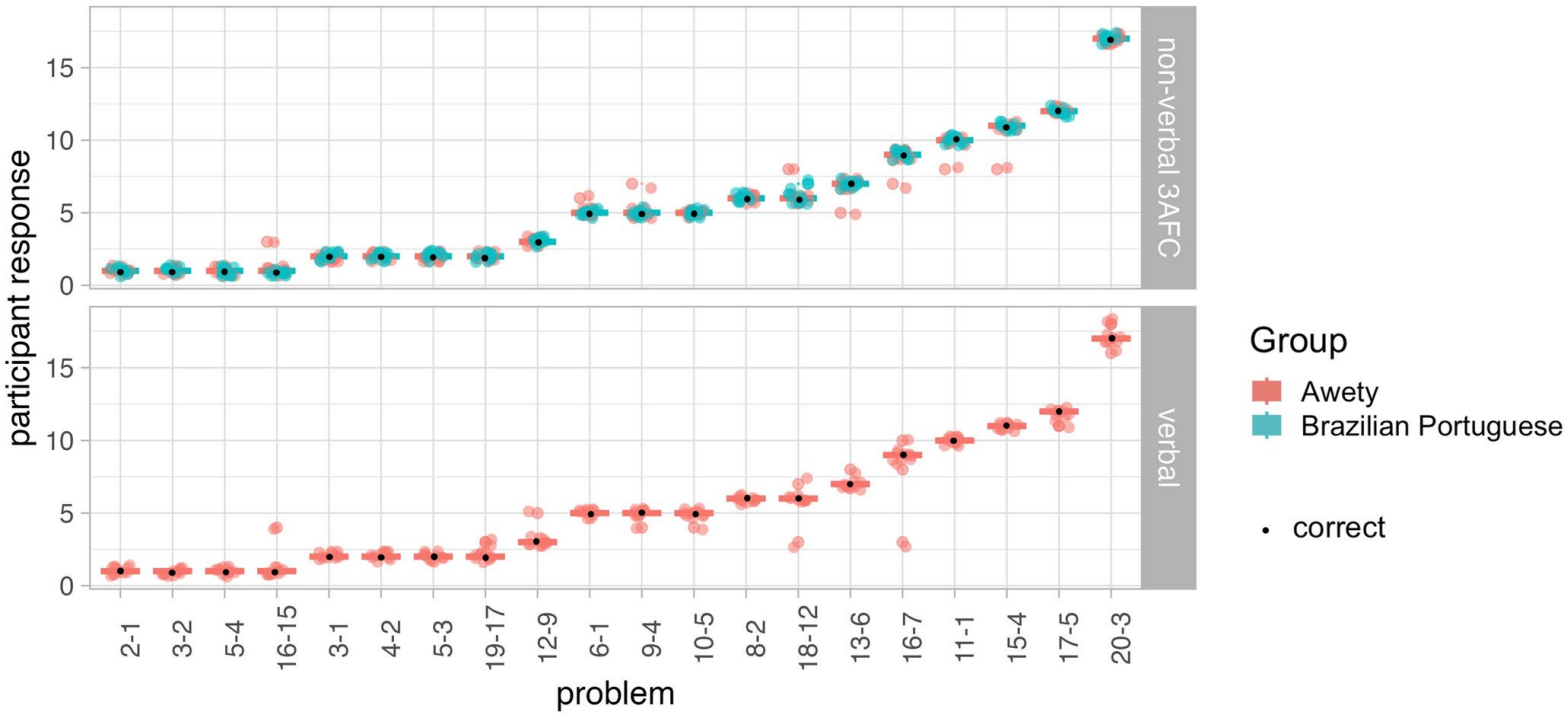
Box plots of responses to the subtraction tasks given by Awetý (red) and Brazilian Portuguese (blue) speakers, with individual responses (dots) in non-verbal (left) and verbal (right) conditions. Only Awetý speakers were tested for verbal subtraction, as it was deemed uninformative for Brazilian Portuguese speakers

As can be seen in Table 3, both Awetý and Brazilian Portuguese speakers performed with high accuracy on the tasks that required non-verbal answers (see Fig. 6), including the subtraction task (Fig. 7). On average, Awetý speakers performed below 95% accuracy on only three out of the eight tasks with non-verbal answers: visual matching for the non-linear display in random order, in percentage (*M*_Awetý_ = 94.6, *SD*_Awetý_ = 4.0) and both versions of the delayed audio-visual matching task (ascending *M*_Awetý_ = 91.7, *SD*_Awetý_ = 9.4; random *M*_Awetý_ = 87.5, *SD*_Awetý_ = 10.6). The mean accuracy of Brazilian Portuguese speakers was above 95% for all tasks except for the delayed audio-visual matching tasks with random order presentation (*M*_Brazilian Portuguese_ = 93.2, *SD*_Brazilian Portuguese_ = 11.9). None of the group comparisons reached statistical significance after correction for multiple comparisons.

The corresponding Bayes factors (BF₁₀ range: 1.186–2.244) fall within the range conventionally considered inconclusive (0.33 < BF₁₀ < 3) indicating limited sensitivity of the present data to distinguish between the null and alternative hypotheses. Observed mean accuracy was numerically lower for Awetý speakers than for Brazilian Portuguese speakers on the visual matching task with the non-linear display in random order (*M*_Awetý_ = 94.6, *M*_Brazilian Portuguese_ = 97.5, *BF*_*10*_ = 1.531) and on both versions of the delayed audio-visual matching task (ascending *M*_Awetý_ = 91.7, *M*_Brazilian Portuguese_ = 96.8, *BF*_*10*_= 1.186; random *M*_Awetý_ = 87.5, *M*_Brazilian Portuguese_ = 93.2, *BF*_*10*_ = 2.244). These differences should therefore be interpreted cautiously. In addition, Awetý speakers showed high accuracy levels on the two cardinality tasks with non-verbal responses (How many *M*_Awetý_ = 97.1, *SD*_Awetý_ = 5.0; Give-N *M*_Awetý_ = 97.3, *SD*_Awetý_ = 3.4).

#### Verbal answers

Awetý speakers responded with high accuracy when they were asked to respond with spoken number words, whether this was for subtraction (*M*_Awetý_ = 92.2, *SD*_Awetý_ = 8.1; see Fig. 7), in the how many task (*M*_Awetý_ = 95.0, *SD*_Awetý_ = 5.5), or in verbal counting (100% counted up to 20).

**Fig. 8:**
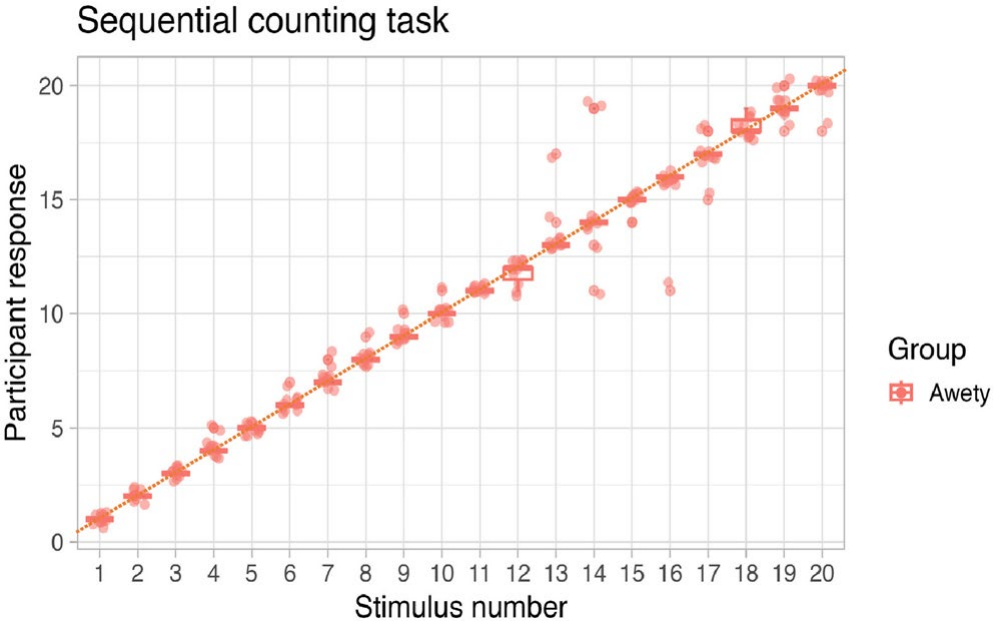
Box plots of verbal responses given by Awetý (red) speakers in the sequential counting task, with individual responses (dots)

The accuracy of the Awetý speakers was lower than in other tasks when they were presented with a series of auditory clicks and they had to count those clicks and respond with a spoken number (*M*_Awetý_ = 86.4, *SD*_Awetý_ = 14.8) (see Fig. 8). However, this is largely due to one individual who made a large number of mistakes on this task (*M* = 45). When this participant was taken out, the mean accuracy of the remaining Awetý speakers was 90.4% (*SD*_Awetý_ = 6.0).

## General discussion

It has been claimed that Amazonian languages typically have a number inventory of five words or fewer, that their speakers do not use a counting routine, and that their number words are not true numerals denoting cardinal numbers (Aikhenvald, 2022: 436–437). However, it is important to note that Amazonia is a region of exceptional linguistic diversity (Aikhenvald, 2015), and number systems vary widely across its languages (Silva Sinha et al., 2017). While Pirahã and Mundurukú have attracted attention due to their very limited numerical lexicons, they are not necessarily representative of Amazonian languages. Awetý, with a system of 20 number terms, offers an important source of comparative evidence within this typological landscape. Awetý has previously been described as having number words only up to ten, and it has been claimed that although Awetý people can count to 10 if prompted, the numbers from 5 to 10 are not lexicalised and are not commonly used (Drude, 2009, reported in Chan, 2024). We found, to the contrary, that Awetý speakers use indigenous complex polymorphemic expressions to count to 20 (Silva Sinha & Sabino Kamaiurá: Numeral construction and embodied quantification in the Awetý language, submitted), that they use these terms with consistency, and people are able to perform number tasks accurately. As might be expected from previous research (e.g., Pica et al., 2004), Awetý participants were able to accurately discriminate larger from smaller approximate numbers. Both Awetý and Brazilian Portuguese controls showed a ratio effect on accuracy. The only difference between the groups was that as the ratio became harder to discriminate, Awetý—but not Brazilian Portuguese speakers were slower to respond, but no less accurate. These results are consistent with accounts that approximate large number discrimination reflects a pan-cultural approximate number system that does not depend on symbolic mediation.

Awetý participants were also accurate in their counting routine performance (“Highest Count”, in which they simply recited the number words in ascending order) up until 20. Both the existence of a counting routine in Awetý, and the lexicalization of the concept of counting in the verb *ipapat*, challenge claims of a consistent “lack of a counting routine” in Amazonian languages (Aikhenvald, 2022: 436). Notably, however, Awetý participants stopped counting at exactly 20.^2^ Crucially, Awetý speakers use their fingers, hands, toes, and feet in counting. The number words also express and represent this embodied counting practice. During the counting of arrays of items, participants first matched the array quantity with their hands and then looked down at their feet to match quantities above ten. This embodied practice was also evident in verbal counting routines, for which participants’ hands, fingers, feet, and toes were referred to. Participants also touched objects and images while counting, and it is likely that these embodied practices helped keep track of items. Although this may seem an intuitive way to keep track of numbers, it is not found universally, as demonstrated in home signers and non-counters of Nicaraguan Sign Language (Spaepen et al., 2011; Flaherty & Senghas, 2011).

Awetý participants were also highly accurate, with very few exceptions, and rarely had an error greater than one in their verbal number comprehension. They were able to accurately perform matching of arrays of up to 20 items (see Fig. 8), in different modalities (visual and auditory), presented under different memory demands (simultaneously presented blocks vs. delayed auditory knocks) and in different array orders (ascending vs. random; linear vs. non-linear). These results indicate that Awetý speakers can reliably represent and match exact quantities up to at least the maximum of their number inventory (i.e., 20). It remains an open question whether they are able to keep track of larger exact quantities beyond their count range.

No statistically reliable differences between Awetý and Brazilian Portuguese groups were observed. In a small number of tasks (i.e., visual number matching task with non-linear arrays presented in random order and delayed audio-visual number matching tasks), Awetý speakers showed numerically lower accuracy than Brazilian Portuguese speakers. However, the corresponding Bayes factors fell within the inconclusive range, and we are not able to draw conclusions about group differences. Given the small sample size and overall high accuracy, these patterns should be interpreted with caution. Although we did not observe reliable group differences, variability across individuals raises the question of the possible role that formal education plays in numerical abilities. While Brazilian Portuguese group have typically undergone structured formal schooling, which includes extensive training in arithmetic and number concepts, the Awetý community has limited access to such formal schooling. Instead, many Awetý participants attend indigenous schools, which focus on culturally relevant education rather than the structured, formal curriculum found in non-indigenous settings. A relationship between schooling and the approximate number system has been found in the Indigenous Mundurucu society (Piazza et al., 2013), and more recently, a meta-analysis of number knowledge among children and adult speakers of Tsimane’ found that education was critical for children to be able to count: no 3–8 year old child could count fully, unless they had experienced formal schooling (O’Shaughnessy et al., 2023). However, the same study also found that more than half Tsimane’ adults could count even without any formal schooling, suggesting other factors, such as participating in a market economy, may be sufficient for full counting (O’Shaughnessy et al., 2023). It is possible, then, that the combination of limited formal schooling and other culturally embedded learning experiences within the Awetý community was sufficient to scaffold the development of those numerical abilities observed here.

It is also important to acknowledge that many of the Awetý participants in this study are bilingual, familiar with both Awetý and Brazilian Portuguese number systems, and some also spoke Kamaiurá, another Indigenous language in the region, which—like Awetý—has a lexicalized number system extending to 20 (Seki, 2000). Bilingualism is known to influence cognitive processes, including numerical cognition (e.g., Cerda & Wicha, 2024; Peng et al., 2020; Wagner et al., 2015). In the case of the Awetý speakers, exposure to number words in other languages might have influenced their performance on the numerical tasks, making it difficult to isolate the specific impact of Awetý number words on putatively non-linguistic tasks. As Brazilian Portuguese has a fully elaborated decimal system extending beyond this range, whereas Awetý counting is limited to twenty, differences in lexical resources could conceivably influence performance on larger exact quantities. However, all tasks were administered in Awetý, and participants consistently responded using Awetý number words. While it is possible that multilingual knowledge of numerals across languages may reinforce conceptual understanding, there was no visible evidence of cross-linguistic interference or code-switching during the tasks. There were, however, not a sufficiently large number of participants to be able to tease apart the specific contributions that bilingualism or formal schooling may play in the numerical abilities of the Awetý.

Finally, we note the possible role of cultural context of number use in numerical cognition. The relevance and use of numbers in daily life appear to differ in interesting ways between the Awetý and Brazilian Portuguese speakers communities. While the Brazilian Portuguese speakers in Brazil live in a market-integrated culture where formal schooling is the norm and certain professions rely heavily on an exact number knowledge, the Awetý have distinct cultural practices. The Awetý number terminology expresses an embodied way of engaging with numbers, perhaps reflecting the way that numbers are culturally embedded in physical and social practices. The extent to which large exact numbers are culturally relevant to the Awetý is not clear. This leaves open the possibility that the two groups may differ in how they process quantities larger than 20. This is a matter for future studies to explore.

To conclude, this study demonstrates that Awetý speakers, whose number system has previously been described as limited to five or ten terms (Chan, 2024), reliably use a counting system extending to twenty and are capable of performing exact numerical tasks across both verbal and non-verbal formats within this range, including counting, matching, and basic arithmetic operations. Future research should explore how Awetý speakers handle cardinalities beyond 20, where no indigenous linguistic number terms are available, in order to further examine how language, culture, and numerical practices interact to shape numerical cognition beyond the attested counting range.

## Data Availability

The field manual and anonymized data are publicly available via the Open Science Framework: [https://osf.io/7hguz/files/osfstorage](https:/osf.io/7hguz/files/osfstorage).

## Appendix

**Table 4:** Subtraction items with correct answers and distractors

Subtraction item	Correct	Distractors
20 − 3	17	14, 15
8 − 2	6	4, 8
15 − 4	11	7, 8
9 − 4	5	4, 7
17 − 5	12	9, 10
6 - 1	5	4, 6
12 − 9	3	1, 4
3 − 1	2	3, 5
16 − 7	9	6, 7
3 − 2	1	3, 4
19 − 17	2	4, 5
5 − 4	1	2, 4
18 − 12	6	7, 8
4 − 2	2	3, 4
10 − 5	5	3, 4
4 − 3	1	2, 4
13 − 6	7	5, 6
2 − 1	1	3, 4
16 − 15	1	4, 3
5 − 3	2	1, 4
11 − 1	10	8, 9

**Table 5:** Target items and the response options for the How-many task with non-verbal responses

Target item	Response options
18	17, **18**, 20
10	8, **10**,12
15	13, **15**, 17
4	2, 3, **4**
2	**2**, 3, 4
19	18, **19**, 20
6	4, **6**, 7
8	7, **8**, 9
13	8, 10, **13**
11	9, **11**, 14
14	12, **14**, 16
12	7, 9, **12**
17	13, 15, **17**
9	7, 8, **9**
1	**1**, 2, 4
16	14, **16**, 18
20	15, 18, **20**
3	1, 2, **3**
7	**7**, 8, 10
5	**5**, 6, 8

## Footnotes

1 The morphological analysis is based primarily on the knowledge of one of the authors [WKS] who is an Indigenous knowledge holder and native speaker of Awetý and who holds a doctorate in linguistics.

2 We did not formally assess Awetý participants’ knowledge of Brazilian Portuguese number words. Ethnographic observations and general knowledge of local language practices suggests that the use within the language community of Brazilian Portuguese number words beyond 20 is not common, but larger numbers may be employed in contexts of education and/or cultural and linguistic contact.

## Declarations

### Open practices statement

Open practices statement: The study was not preregistered. The field manual and anonymized data are publicly available via the Open Science Framework: https://osf.io/7hguz/files/osfstorage.
